# Bimodal high-affinity association of Brd4 with murine leukemia virus integrase and mononucleosomes

**DOI:** 10.1093/nar/gku135

**Published:** 2014-02-11

**Authors:** Ross C. Larue, Matthew R. Plumb, Brandon L. Crowe, Nikoloz Shkriabai, Amit Sharma, Julia DiFiore, Nirav Malani, Sriram S. Aiyer, Monica J. Roth, Frederic D. Bushman, Mark P. Foster, Mamuka Kvaratskhelia

**Affiliations:** ^1^Center for Retrovirus Research and College of Pharmacy, The Ohio State University, Columbus, Ohio 43210, USA, ^2^Department of Chemistry and Biochemistry, The Ohio State University, Columbus, Ohio 43210, ^3^Department of Microbiology, Perelman School of Medicine, University of Pennsylvania, Philadelphia, PA 19104, USA and ^4^Department of Pharmacology, Robert Wood Johnson Medical School, University of Medicine and Dentistry of New Jersey, Piscataway, NJ 08854, USA

## Abstract

The importance of understanding the molecular mechanisms of murine leukemia virus (MLV) integration into host chromatin is highlighted by the development of MLV-based vectors for human gene-therapy. We have recently identified BET proteins (Brd2, 3 and 4) as the main cellular binding partners of MLV integrase (IN) and demonstrated their significance for effective MLV integration at transcription start sites. Here we show that recombinant Brd4, a representative of the three BET proteins, establishes complementary high-affinity interactions with MLV IN and mononucleosomes (MNs). Brd4(1–720) but not its N- or C-terminal fragments effectively stimulate MLV IN strand transfer activities *in vitro*. Mass spectrometry- and NMR-based approaches have enabled us to map key interacting interfaces between the C-terminal domain of BRD4 and the C-terminal tail of MLV IN. Additionally, the N-terminal fragment of Brd4 binds to both DNA and acetylated histone peptides, allowing it to bind tightly to MNs. Comparative analyses of the distributions of various histone marks along chromatin revealed significant positive correlations between H3- and H4-acetylated histones, BET protein-binding sites and MLV-integration sites. Our findings reveal a bimodal mechanism for BET protein-mediated MLV integration into select chromatin locations.

## INTRODUCTION

In order to replicate, retroviruses must integrate reverse transcribed viral DNA into the host chromosome. The distributions of integrated proviruses in host genomes are not random and vary markedly for different retroviral genera. Two well-known examples include the lentiviruses, such as human immunodeficiency virus type 1 (HIV-1), which favors integration at active transcriptional units and the gammaretroviruses, such as murine leukemia virus (MLV), which favors integration at transcription start sites (1–3). The primary viral factor controlling the distribution of retroviral integration sites is integrase (IN), whose key role was demonstrated using a chimeric HIV-1 virus with its IN sequence replaced with the MLV counterpart (3). Integration sites of the chimeric virus significantly changed from active genes towards transcription start sites and thus trended closer to MLV than HIV-1.

Studies of the mechanism of HIV-1 integration have elucidated a cofactor, the cellular chromatin binding protein LEDGF/p75, which acts as a tether to chromatin (4–10). The LEDGF/p75 C-terminal region, termed the Integrase Binding Domain or IBD, binds lentiviral INs (10,11). The N-terminal portion of LEDGF/p75, which contains a PWWP domain, nuclear-localization signal, AT hooks and highly charged regions, selectively associates with chromatin. We have recently shown that the PWWP domain cooperatively engages both the trimethylated H3 tail (H3K36me3), a hallmark of active genes and DNA wrapped around nucleosomes to ensure high-affinity binding of LEDGF/p75 with chromatin (12).

Recent efforts have also focused on exploring the molecular mechanisms of MLV-integration-site selectivity. The significance of these studies is exemplified by the development of MLV-based vectors for human gene-therapy of primary immunodeficiencies. This therapeutic concept was first successfully demonstrated for hematopoietic stem cell (HSC) gene-therapy for X-linked severe combined immune deficiency (SCID-X1) (13). In separate clinical trials from 1999 to 2009, a total of 20 SCID-X1 patients underwent treatment for a gene defect in interleukin 2 common gamma chain using MLV-based HSC gene-therapy (14). Unfortunately, 5/20 of the patients have since developed leukemia (14). The associated cancer in these patients was linked to the insertion of MLV-based vectors near the *LMO-2* and *CCND2* proto-oncogenes, where integration resulted in transcriptional upregulation of proto-oncogenes (15–17). In separate studies for the treatment of different genetic immunodeficiencies such as Wiscott–Aldrich syndrome (WAS) and X-linked chronic granulomatous disease (CGD), patients likewise have developed cancer (18–20). These studies have highlighted the importance of integration site selection by MLV-based vectors on the outcomes of gene-therapy.

We have previously identified BET proteins (Brd2, 3 and 4) as the principal binding partners of MLV IN and demonstrated their significance for targeting MLV integration to transcription start sites (21). More recent reports have corroborated these findings (22,23) as well as earlier yeast 2-hybrid experiments which showed interactions between MLV IN and Brd2 (24). BET proteins (Brd2, 3 and 4) are part of the BET protein family (Brd2, 3, 4 and T) and the extended BET family, which also includes Brd1, 7, 8 and 9. While BrdT is only expressed in the testis, the other BET proteins are ubiquitously expressed and have been implicated in control of the cell cycle, transcription and DNA replication [reviewed in (25,26)]. BET proteins exhibit dual N-terminal bromodomains (BD-I and BD-II), conserved motifs termed ‘A’ and ‘B’, basic residue-enriched interaction domain (BID), C-terminal extra-terminal (ET) domain and SEED domain, which contains glutamic and aspartic acid residues interspersed between polyserine residues. The bromodomains are known to bind acetylated H3 and H4 tails on chromatin (27,28), whereas the ET and SEED domains associate with a variety of cellular proteins including chromatin-modifying factors, transcription factors, histone modification enzymes, as well as interact with a number of viral proteins [reviewed in (26)]. The BID domain has been shown to control intra- and inter-molecular interaction of Brd4 with cellular factor p53 (29). While only single isoforms are known for Brd2 and 3, Brd4 is expressed in two isoforms—Brd4(aa 1–720) and Brd4(aa 1–1362). The longer Brd4 isoform contains an additional C-terminal motif of ∼38 amino acids that has been implicated in several protein–protein interactions and potentially plays a role in HIV-1 latency (30,31).

This study aims to clarify how BET proteins recognize MLV IN and direct MLV integration to specific chromatin sites. Like most retroviral INs, MLV IN comprises three domains: the N-terminal domain (NTD), the catalytic core domain (CCD) and the C-terminal domain (CTD). The NTD contains the NTD-extension domain (NED) (32) and the Zn-binding motif (HH-CC type) and is thought to help IN multimerization. The CCD contains the enzyme active site which is characterized by a three amino acid triad (DDE) that coordinates Mg^2+^ and is responsible for 3′ processing and strand-transfer activities. The CTD is involved in multiple functions including binding DNA and could also be engaged in interactions with other proteins including the BET proteins (21).

Here we have used mass spectrometry (MS)-based protein footprinting and NMR to examine interactions between recombinant purified Brd4 and MLV IN. Our studies have mapped the interacting interfaces between the C-terminal Brd4 ET amino acids and the 10 amino acid segment at the C-terminus of MLV IN. Furthermore, we show that Brd4 interacts with high affinity with native mononucleosomes (MNs). These interactions are mediated by the Brd4 N-terminal fragment, consisting of two bromodomains and conserved motifs A and B, which engages both acetylated histone peptides and DNA wrapped around the nucleosomes. Collectively, our studies reveal a bimodal mechanism for BET proteins-mediated targeting of MLV integration to modified chromatin sites.

## MATERIALS AND METHODS

### Plasmids and cloning

The human 6xHis–Brd4(1–720), GST–MLV IN and 6xHis–MLV IN clones were previously described (21,33). The following truncated motifs/domains were PCR amplified from Brd4(1–720) and subcloned into pEX-N-His (Origene): nBrd4(1–461), cBrd4(462–720), Brd4 B/BID(462–599), Brd4 ET(600–678) and Brd4 ET/SEED(600–720). Cloning of the following was performed using a site-directed mutagenesis kit (Stratagene); truncated mutants of GST-MLV IN and 6xHis–MLV IN were generated with a stop codon at amino acids 393 and 399.

### Expression and purification of recombinant proteins

All proteins were expressed in *Escherichia coli* BL-21(DE3) cells and induced for 3.5 h with 1 mM IPTG at 37°C (Brd4 constructs) or 30°C (MLV IN constructs). 6xHis–Brd4(1–720) and GST–MLV IN as well as their truncation mutants, were purified as previously described (21). 6xHis–MLV IN was purified as follows; the cells were lysed in [750 mM NaCl, 50 mM HEPES pH 7.5, 7.5 mM CHAPS, 10% glycerol, 2 mM β-mercaptoethanol (BME) and 20 mM imidazole] and proteins were purified using a Ni-NTA Nickel column (GE Healthcare) with a 60-mM imidazole wash in the same buffer and eluted with 500 mM imidazole in the same buffer. [U-^15^N]–6xHis–Brd4(600–678) was grown in M9 minimal medium supplemented with 1% (v/v) Eagle Basal Vitamin Mix (Life Technologies) with 1 g/l of ^15^N-ammonium chloride (Cambridge Isotope Laboratories) as the sole nitrogen source. Cells were induced with 1 mM IPTG overnight at 30°C and purified as described previously (21).

### Homogenous time-resolved fluorescence and affinity pull-down-based assays

MLV IN strand transfer activities were assayed using the homogenous time-resolved fluorescence (HTRF)-based method (21). A synthetic 5′-biotinylated 40-bp SMYD1 DNA fragment [as described in (12)] was used to monitor DNA binding to Brd4(1–720) and its truncation mutants by HTRF-based and affinity pull-down assays. HTRF-based assays used purified proteins and DNA substrates at the following final concentrations: 6xHis–Brd4(1–720) and its truncated fragments, 125 nM, and DNA, 10 nM. All substrates were diluted and mixed in binding buffer [150 mM NaCl, 2 mM MgCl_2_, 0.1% NP40, 1 mg/ml BSA (New England Biolabs), 25 mM Tris pH 7.4]. After 30 min incubation, 40 nM anti-6His XL665 (CisBio) and 3 nM europium-labeled streptavidin (Perkin Elmer) in binding buffer, was added to the reaction followed by 3 h incubation at 4°C. Samples were transferred to a white 384-well plate and read on an Enspire Perkin Elmer machine. DNA pull-down assays were performed using the same DNA substrate and 6xHis–Brd4(1–720) and ultralink immobilized streptavidin beads (Pierce). Reactions were performed in 50 mM HEPES pH 7.5, 100 mM NaCl, 0.5% NP40, 2 mM BME with constant DNA (8.3 µM) and variable concentrations of Brd4(1–720) (16, 8, 4, 2 and 1 µM, 500, 250 and 125 nM). Pull-down efficiencies were determined by SDS-PAGE and Coomassie staining.

Interactions between MLV IN and Brd4 were monitored by affinity pull-down assays as described in Sharma *et al.* (21) with detection by western blotting with anti-His antibody (Abcam). Protein pull-downs were performed with glutathione sepharose 4B beads or Ni sepharose 6 Fast flow beads (both from GE Healthcare). GST–MLV IN was used to pull-down 6xHis–Brd4(1–720) or its truncation mutants in 50 mM HEPES pH 7.5, 100 mM NaCl, 0.5% NP40, 2 mM BME, 1x complete protease cocktail (Roche). Titrations were performed with the following concentrations (500, 250, 125, 62.5, 31.25, 15.6 and 7.8 nM) for 6xHis–Brd4(1–720) and 6xHis–cBRD4(462–720) and (2000, 1000, 500, 250, 125, 62.5 and 31.25 nM) for 6xHis–Brd4 ET(600–678) and 6xHis–Brd4 ET/SEED(600–720).

Native MNs were purified as previously described (12). 6xHis–Brd4(1–720) or its truncation mutants were used to pull-down native MNs in 50 mM HEPES pH 7.5, 200 mM NaCl, 0.5% NP40, 2 mM BME, 1x complete protease cocktail and 20 mM imidazole. Titrations were performed with the following concentrations (500, 250, 125, 75, 37.5, 18.75 and 9.4 nM) with detection by standard western-blotting procedures and rabbit polyclonal histone H3 antibody (Abcam). Titrations were quantified using ImageJ software and *K_d_*s were determined by fitting the data with the Hill equation using Origin8 software (OriginLab).

### MS-based protein footprinting

Protein footprinting assays were carried out as described (34,35). MS-based protein footprinting allows one to compare the surface topologies of proteins in their free form versus in bound complexes using small amino acid-selective chemical modifiers such as *N*-hydroxysuccinimidobiotin (NHS-Biotin). Surface accessible Lys residues that are readily modified by NHS-biotin in unliganded protein but are shielded from the modification by the bound partner are identified by MS analysis. Pull-down-based assays were used to enrich the complex formation using glutathione sepharose 4B beads and GST–MLV IN and 6xHis–Brd4(1–720). Both GST–MLV IN and 6xHis–Brd4(1–720) were modified in the presence or absence of the other. Individual proteins or protein–protein complexes were subjected to modification with 1 mM sulfo-N-hydroxysuccinimide (NHS)-biotin (Pierce) and then separated by SDS-PAGE. Individual protein bands were excised, subjected to in-gel proteolysis by trypsin and the resulting peptides were analyzed using an AXIMA-CFR MALDI-ToF instrument and α-cyano-4-hydroxy-cinnamic acid as the matrix.

### NMR

[U-^15^N]–6xHis–Brd4 ET(600–678) was concentrated by ultrafiltration to 190 μM in buffer containing 20 mM Tris pH 7.0, 100 mM NaCl, 1 mM DDT and 0.02% NaN_3_. D_2_O was added to 5% (v/v) and DSS to 0.5 mM. NMR spectra were recorded at 25°C on an 800-MHz Bruker Avance DRX spectrometer equipped with a cryogenically cooled triple-resonance single-axis gradient probe. Data were processed with NMRPipe (36) and analyzed with NMRViewJ. An NMR titration was performed with unlabeled 6xHis–MLV IN CTD(329–408) being added to [U-^15^N]–6xHis–Brd4 ET(600–678). In addition, titrations were also performed with a synthetized peptide consisting of 17 amino acids (aa 389–405) at the C-terminal tail of MLV IN (Biomatik). Four 2D ^1^H-^15^N correlation spectra were recorded containing 6xHis–Brd4 ET(600–678) and 0, 0.5, 1 and 1.5 equivalents of 6xHis–MLV IN CTD(329–408). The chemical shift perturbations (CSP) were determined using the following equation: 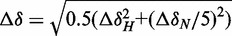 (37). Amide resonances from 95% of the non-proline residues could be assigned by correspondence to those reported for the mouse Brd4 ET (38), whose sequence is identical to its human counterpart. Our human Brd4 ET construct also contained an additional 16 amino acids on the N-terminal due to the 6xHis tag and TEV protease site and two differing residues on the C-terminus, which replaced the last four non-ET residues in the mouse Brd4 ET construct as a result of the sub-cloning. Due to sequence differences outside of the ET region, the residue numbers for Brd4(1–720) human protein is obtained by adding 592 to the sequence in the deposited coordinates and assignments (38). The sidechain H^ε^ of Arg 666 was also observed and assigned. In addition to the expected resonances from the Brd4 ET, six backbone amide resonances and two sets of sidechain Gln or Asn amide signals were observed and were attributed to the N- and C-terminal differences and were not assigned.

### Bioinformatics and statistical analyses

Our previous study (21) described the MLV-integration-site data for HEK293T cells treated with 500 nM JQ-1 inhibitor or DMSO as well as for HEK293T cells transfected with scrambled siRNA (indicated as ‘Sci’) or a pool of Brd2, 3 and 4 siRNAs [indicated as ‘Brd(2+3+4)’]. For comparison, published integration sites of Avian sarcoma leukosis virus (ASLV), HIV-1 and MLV were also analyzed (5,39–41). The histone post-translational modifications used in the analysis have been previously described (42). The receiver operator characteristic (ROC) curve-area method was used to quantify the relationship between the integration-site frequencies relative to matched random controls for each of the annotated features as described previously (42,43). Each tile in the heatmap depicts the ROC value of each comparison. The statistical methods and tests used to determine whether the calculated ROC areas were significantly different from one another or from 0.5 (matched random controls) were described previously (42,43).

## RESULTS

### Brd4(1–720) is required for effective stimulation of MLV integration *in vitro*

**Figure 1.**
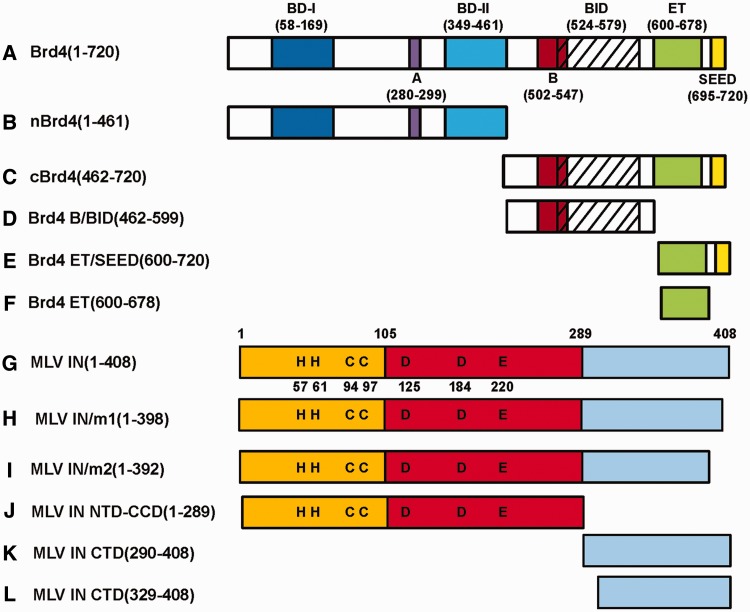
Brd4 and MLV IN constructs used in the present study. All Brd4 constructs (**A**–**F**) contained the N-terminal hexa-histidine tag, whereas MLV IN proteins (**G**–**L**) contained either GST- or hexa-histidine tag at their N-terminus.

In order to understand how BET proteins modulate MLV IN activities we studied the protein constructs depicted in Figure 1. The BET proteins (Brd2, 3 and 4) share a high degree of similarity in both sequence and domain organization. We found that recombinant Brd4(1–720) was the most amenable to expression and purification and used it in our studies. We have recently reported that the addition of 0.5 µM Brd4(1–720) stimulated the strand-transfer reaction catalyzed by MLV IN *in vitro* (21). A more recent report (22) tested recombinant ET domains of BET proteins and showed that this isolated domain can also stimulate MLV IN activities, albeit only at relatively high concentrations (24 µM) compared to Brd4(1–720) (described below).

**Figure 2.**
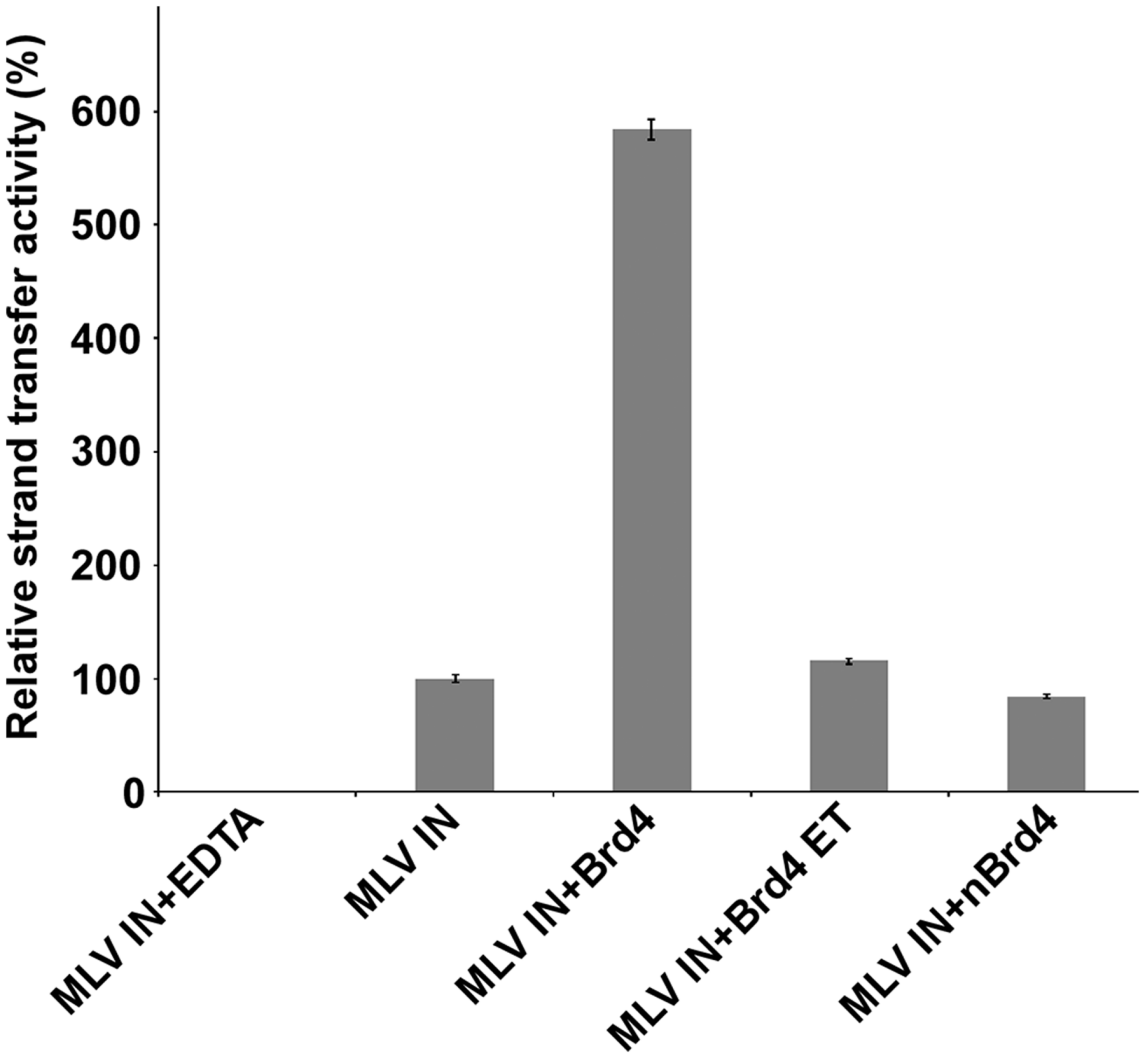
Effects of Brd4(1–720), nBrd4 and Brd4 ET on MLV IN strand transfer activities. HTRF-based strand-transfer assays (21) were performed for accurate quantitative and comparative analyses with HTRF signals plotted. Where indicated 0.5 µM 6xHis–Brd4(1–720), 6xHisn–Brd4(1–461) or 6xHis–Brd4 ET(600–678) was added to the reaction containing 0.3 µM GST–MLV IN. As a control, 50 mM EDTA was added to a reaction containing both 6xHis–Brd4(1–720) and GST–MLV IN. Bars represent means ± SD [*n* = 3].

We examined the effects of addition of 0.25, 0.5, 1.0 and 2.0 µM Brd4(1–720), nBrd4 (the N-terminal fragment of Brd4, Figure 1) and Brd4 ET on MLV IN (0.3 µM) strand transfer activities. Brd4(1–720) but neither 6xHis–nBrd4(1–461) nor 6xHis–Brd4 ET(600–678) enhanced MLV IN strand transfer activity at the concentrations tested (for clarity only the representative 0.5 µM data is depicted in Figure 2). It should be noted that in these assays naked double-stranded DNA, which does not contain any histones, served as the target DNA substrate suggesting that Brd4 can directly bind DNA. Furthermore, the requirement for Brd4(1–720) for potent stimulation of the strand transfer activities suggest that Brd4 may act as a bimodal tether by establishing complementary interactions using its N- and C-terminal portions with target DNA and MLV IN. To examine these possibilities we have carried out biochemical and biophysical analysis of Brd4 binding to MLV IN, DNA and MNs.

### The C-terminal segment of MLV IN is necessary and sufficient for high-affinity interaction with Brd4

We next mapped the interacting interfaces between Brd4 and MLV IN. Since we previously showed that MLV CTD is responsible for binding to Brd3, we hypothesized the same would hold true for Brd4. However, more recent mutagenesis studies reported that the substitution of certain amino acid residues in the CCD of MLV IN impaired its interaction with BET proteins (22). We thus compared the binding affinities of Brd4(1–720) to truncated constructs of MLV IN (Table 1) and found that full-length GST–MLV IN(1–408) and GST–MLV IN CTD(290–408) bound to 6xHis–Brd4 with very similar affinities, whereas no appreciable binding was detected with the two domain MLV NTD–CCD(1–289) construct. These results indicate that MLV IN CTD is both necessary and sufficient for high-affinity binding to Brd4.

**Table 1.** Binding dissociation constants of 6xHis–Brd4(1–720) with GST–MLV IN domains

	*K_d_* (nM)
MLV IN (1–408)	58.37 9.57
MLV IN NTD-CCD (1–289)	>2000^a^
MLV IN CTD (290–408)	61.23 ± 4.05

Pulled-down 6xHis–Brd4 bands were quantified using ImageJ software and data was fit with the Hill equation.

^a^Binding was not detected at the indicated concentration.

**Figure 3.**
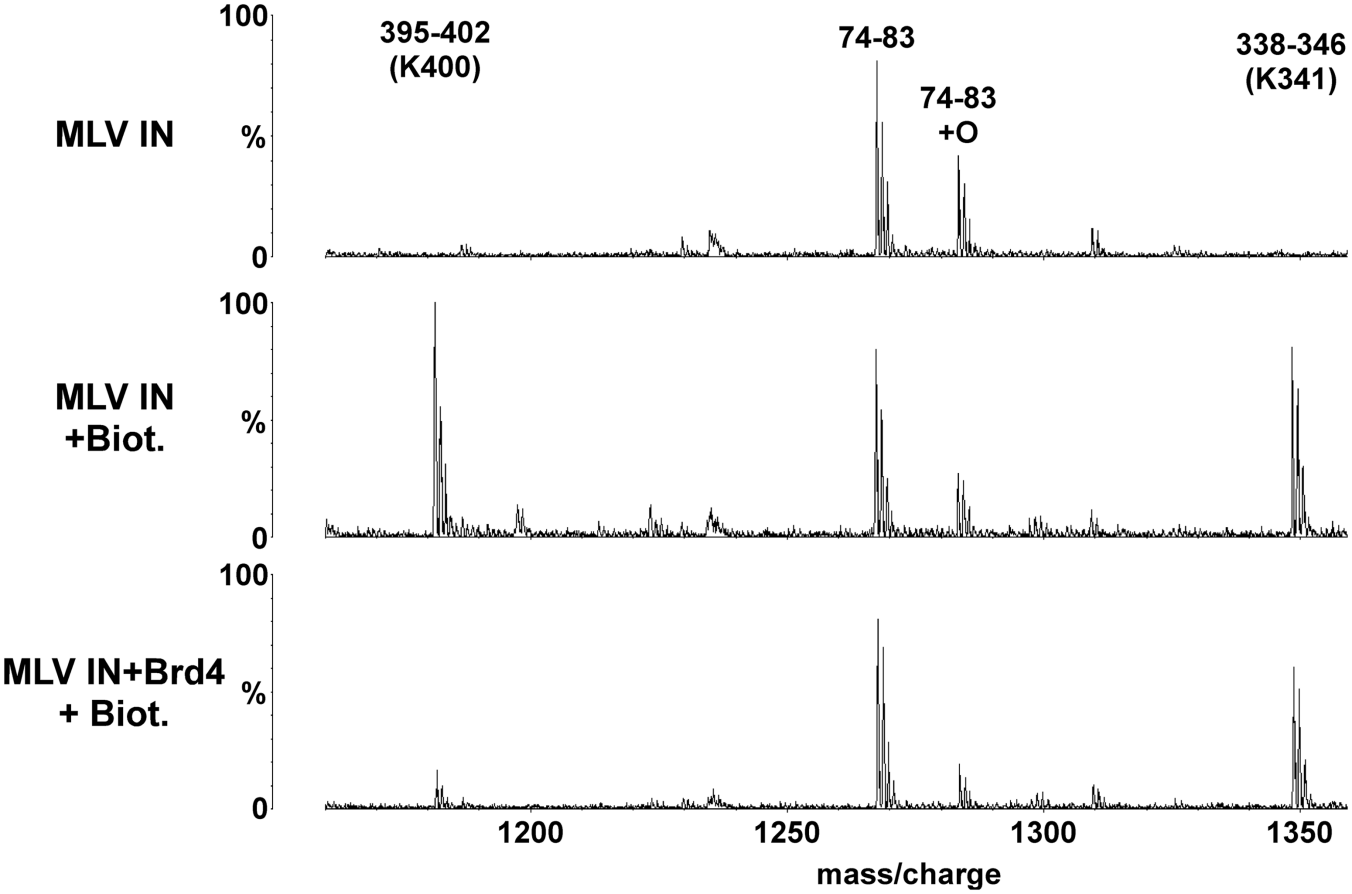
MS-based footprinting of MLV IN interface interacting with Brd4. Representative sections of MALDI-ToF spectra of tryptic peptides of GST–MLV IN are shown. Conditions are indicated to the left with the top profile: GST–MLV IN alone; the middle profile: GST–MLV IN plus treatment with 1 mM NHS-biotin; and the bottom profile: GST–MLV IN preincubated with 6xHis–Brd4(1–720) and then treated with 1 mM NHS-biotin. The start and end amino acid numbers for each identified peak is shown. The Lys residues affected by NHS-biotin modification are indicated in brackets. Also shown are peaks of peptides (74–83) whose intensities do not vary as they do not contain any modified lysine residues allowing them to serve as internal controls.

We next used MS-based protein footprinting to identify the interacting residues in MLV IN (Figure 3). Eight surface lysines (K68, K166, K227, K232, K341, K348, K376 and K400) from each of the three domains of MLV IN reacted with NHS-biotin in the unliganded protein and could be interrogated. Of these, only K400 located at the C-terminal tail of MLV IN was selectively shielded from the modifying agent by bound Brd4 and is shown in Figure 3 along with K341 which was not shielded.

**Figure 4.**
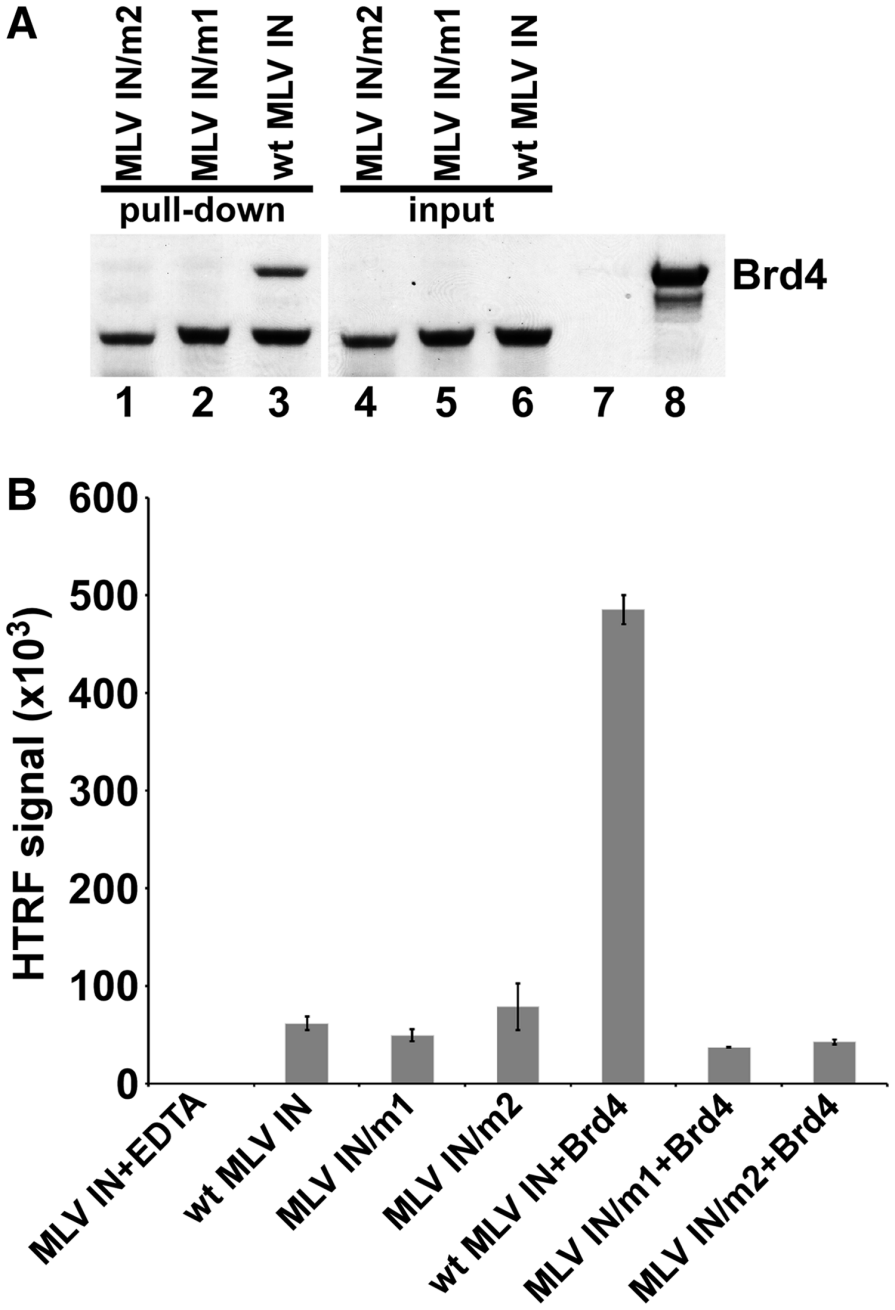
Analysis of MLV IN/m1 and MLV IN/m2. (**A**) Binding of wild type and mutant MLV INs to Brd4. GST-tagged MLV IN/m2 (lane 1), GST-tagged MLV IN/m1 (lane 2) and wild-type MLV IN (lane 3) were used to pull-down 6xHis–Brd4(1–720). Lanes 4–6 show input of mutant and wild-type MLV INs. Lane 7: pull-down of 6xHis–Brd4 in the absence of MLV IN. Lane 8: input of 6xHis–Brd4. (**B**) Comparative HTRF-based strand-transfer activities of wild-type and mutant MLV INs in the presence and absence of 0.5 µM 6xHis–Brd4. As a control, 50 mM EDTA was added to a reaction containing both 6xHis–Brd4 and GST–MLV IN. Bars represent means ± SD [*n* = 3].

We constructed and tested two C-terminally truncated mutant MLV INs–MLV IN/m1 and MLV IN/m2 (Figure 1) by introducing stop codons at amino acid 399 (MLV IN/m1) or 393 (MLV IN/m2), resulting in the removal of the C-terminal 10 or 16 amino acids, respectively. Both truncations thus removed peptides containing K400. Figure 4A shows that wild-type MLV IN but neither mutant MLV INs interacted with 6xHis–Brd4(1–720) in a GST pull-down assay, further establishing the significance of the C-terminal ten amino acids of MLV IN for binding to Brd4.

We next examined the ability of MLV IN/m1 and MLV IN/m2 to catalyze strand transfer activities (Figure 4B), and found that both exhibited wild-type levels. Brd4(1–720) failed to stimulate catalysis by either mutant MLV IN, whereas in control assays Brd4 did stimulate wild-type MLV IN (Figure 4B). Thus, deletion of the C-terminal 10 amino acids of MLV IN compromised stimulation by Brd4.

### Dissecting the Brd4–MLV IN interface

**Figure 5.**
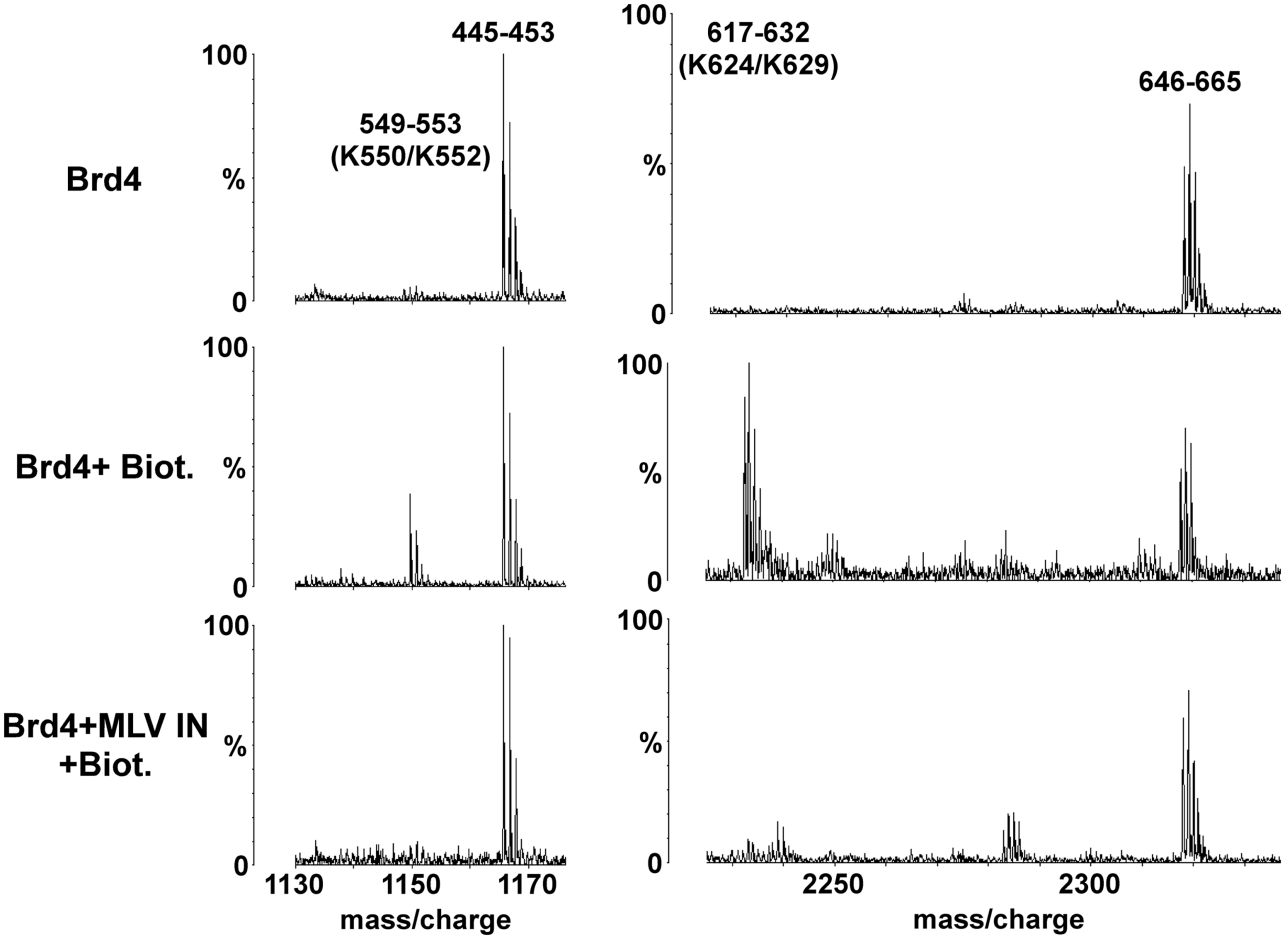
MS-based analyses of interacting interface of Brd4 with MLV IN. Representative sections of MALDI-ToF data for tryptic peptides of 6xHis–Brd4(1–720) are shown. Modification conditions are indicated to the left with the top profile: 6xHis–Brd4(1–720) alone; the middle profile: 6xHis–Brd4(1–720) plus 1 mM NHS-biotin; and the bottom profile: 6xHis–Brd4(1–720) preincubated with GST–MLV IN and then treated with 1 mM NHS-biotin. The peaks are indicated by their start and end amino acid numbers. The Lys residues modified by NHS-biotin are indicated in brackets. Also shown are peaks of peptides (445–453 and 646–665) whose intensities do not vary as they do not contain any modified lysine residues allowing them to serve as internal controls.

**Figure 6.**
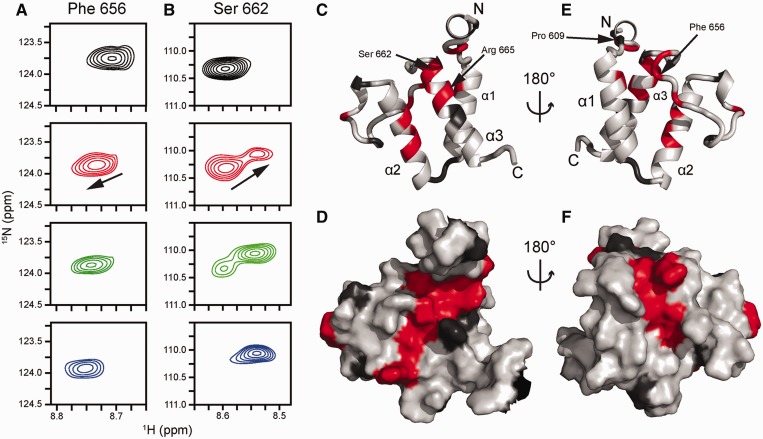
Perturbation of amide NMR signals of Brd4 ET upon titration with MLV IN CTD. (**A**) Amide resonance from F656, which exhibits fast exchange and averaging between the free and bound chemical shifts. (**B**) Amide resonances from S662. This signal exemplifies the slow exchange regime, which is characterized by slow disappearance of the free peak coupled to the appearance of the bound peak. Top (black) spectra, unliganded Brd4 ET; second row (red), Brd4 ET with 0.5 equivalents of MLV CTD; third row (green), Brd4 ET with 1 equivalent of MLV CTD; bottom row (blue), Brd4 ET with 1.5 equivalents of MLV CTD. The black arrow indicates the direction of the CSP from the unliganded to the bound state. Resonance assignments are as previously reported (38). CSPs mapped onto the cartoon (**C**) and surface (**D**) views of the Brd4 ET structure (PDBID: 2JNS) (38) to demonstrate a putative MLV IN-binding interface. Light grey coloring indicates residues whose backbone amide CSP (Supplementary Figure S2) is <0.02 ppm. Dark grey coloring indicates residues with no data either due to being proline or spectral overlap. Red coloring indicates residues whose backbone amide CSP (Supplementary Figure S2) being >0.02 ppm due to MLV IN binding to Brd4 ET which include residues 607, 608, 615, 632, 633, 634, 636, 646, 654, 655, 656, 657, 662, 663, 665, 666 and 667. Specific residues are highlighted for reference.

To identify the Brd4 interface interacting with MLV IN, we performed MS-based protein footprinting (Figure 5), determined the binding affinities (Table 2) of truncated Brd4 derivatives for MLV IN, and used NMR (Figure 6) to elucidate the individual Brd4 amino acids interacting with MLV IN. Using MS-based protein footprinting, we were able to identify 14 Lys residues (K177, K286, K289, K291, K317, K329, K445, K550, K552, K624, K629, K660, K677 and K678) that readily conjugated with NHS-biotin in unliganded 6xHis–Brd4. Of these, K550, K552, K624, K677 and K678 were selectivity shielded from modification after complex formation with GST–MLV IN. These amino acids mapped to the ET domain of Brd4 as well as the BID region which connects the B motif with the ET domain. Representative spectra are shown in Figure 5 with peptides containing the residues of K550/K552 and K624/K629 indicated.

**Table 2.** Binding dissociation constants of various Brd4 fragments with full-length GST–MLV IN

	*K_d_* (nM)
Brd4(1–720)	58.37 ± 9.57
nBrd4(1–461)	>2000^a^
cBrd4(462–720)	77.30 ± 4.21
Brd4 B/BID(462–599)	>2000^a^
Brd4 ET(600–678)	204.97 ± 12.45*
Brd4 ET/SEED(600–720)	205.99 ± 11.58*

Pulled-down 6xHis–Brd4 fragments bands were quantified using ImageJ software and data was fit with the Hill equation.

^a^Binding was not detected at the indicated concentration.

*P < 0.0001 as measured by one-way ANOVA; multiple comparisons of the indicated Brd4 fragments to the Brd4(1–720) control used Dunnett simultaneous test, *n* = 3.

Previously, we had shown that the C-terminal portion of Brd3(420–726) was responsible for MLV IN binding (21). To define the minimal interacting region, we determined the binding affinities of shorter truncated constructs of Brd4 including 6xHis–Brd4 B/BID(462–599), 6xHis–Brd4 ET(600–678) and 6xHis–Brd4 ET/SEED(600–720) (Table 2). 6xHis–Brd4 B/BID(462–599) did not show any detectable binding to GST–MLV IN, whereas very similar *K_d_*s were observed for 6xHis–Brd4 ET(600–678) and 6xHis–Brd4 ET/SEED(600–720). These results indicate that the B motif and the SEED domain are dispensable, while the ET domain is sufficient for the binding of Brd4 to MLV IN. The C-terminal region of 6xHis–cBrd4(462–720) bound GST–MLV IN with slightly higher affinity (*K_d_* of ∼77 nM) compared with 6xHis–Brd4 ET(600–678) (*K_d_* of ∼204 nM). These findings (Table 2) are consistent with MS-based protein footprinting data (Figure 5) showing that in addition to the ET domain, BID domain also engages MLV IN and contributes to higher affinity binding than the ET domain alone.

We next performed NMR-titration experiments using [U-^15^N]–6xHis–Brd4 ET (Figure 6) to identify the Brd4 ET amino acids interacting with MLV IN. Full-length MLV IN was not soluble at the high concentrations required for titration, so we used the more soluble 6xHis–MLV IN CTD(329–408), and monitored the chemical shift perturbations (CSP) of Brd4 ET amide resonances upon titrating MLV IN CTD. Figure 6A and B and Supplementary Figures S1 and 2 reveal that resonances from a number of Brd4 ET residues are perturbed by MLV IN CTD binding. The majority of signals exhibited fast exchange between their free and bound positions, consistent with weak binding (Figure 6A), whereas amides for residues C607, K608 and S662 (Figure 6B) as well as the side chain Hε of R665 showed slow exchange, potentially associated with *cis–trans* isomerization of P609 and/or P661 located nearby. Brd4 ET residues whose amides are affected by MLV IN CTD binding are highlighted in red (Figure 6C, D, E and F) and reveal the putative MLV IN CTD-binding site on Brd4 ET. The majority of these residues are located on Brd4 ET helices 2 and 3 (Figure 6C and D) and the loop connecting these two helices (Figure 6E and F). To delineate the minimal MLV IN segment sufficient for binding Brd4 ET, NMR titrations were also performed with a peptide consisting of 17 amino acids (aa 389–405) at the C-terminal tail of MLV IN. Similar to what was seen with MLV IN CTD, the majority of the same ET residues displayed CSPs upon titration of the peptide. Representative amino acid R665 is shown in Supplementary Figure S3, which exhibited slow exchange between the free and bound positions upon addition of MLV IN CTD or the peptide.

### Brd4 binds DNA and MNs

**Figure 7.**
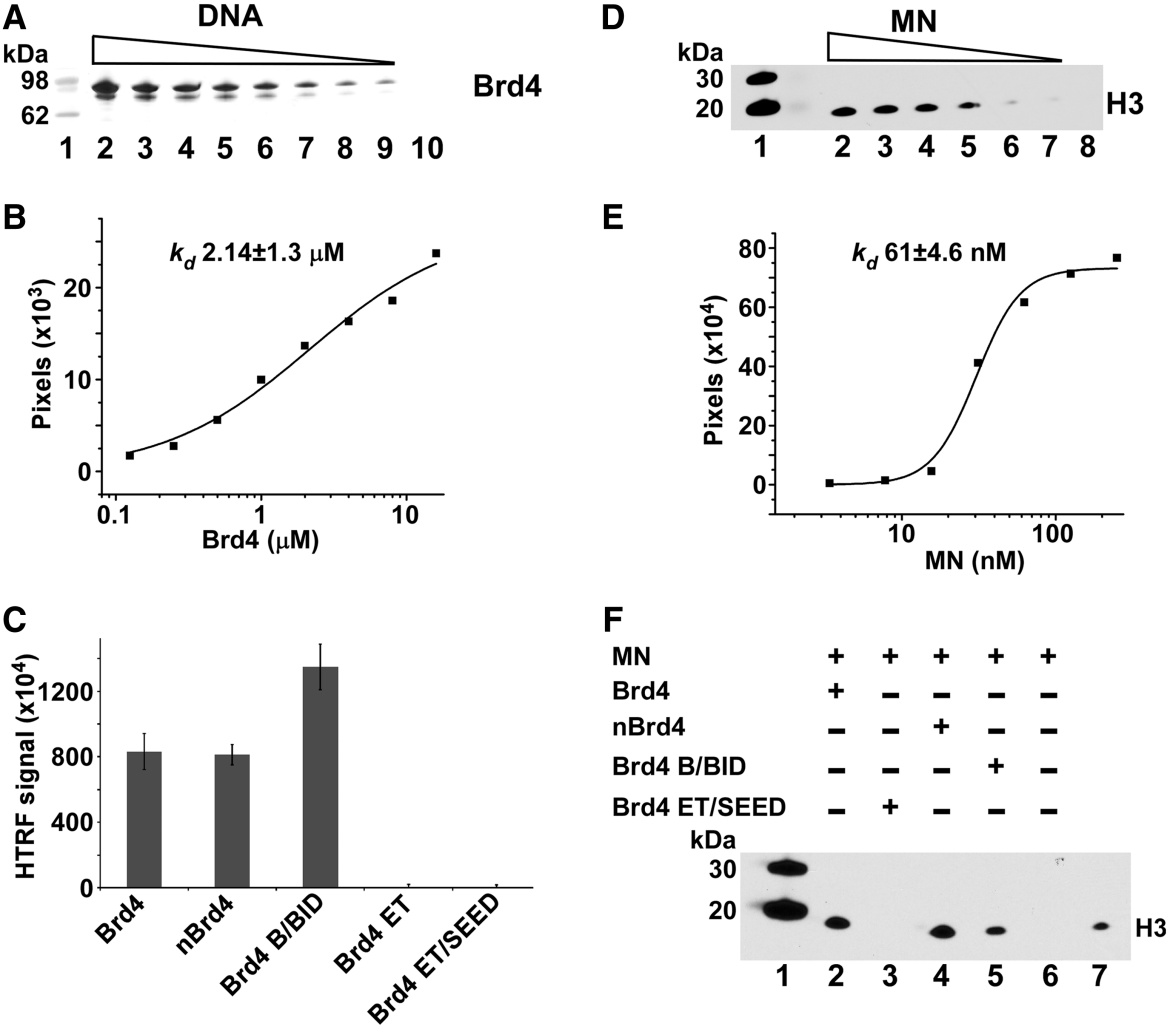
Brd4 interactions with DNA (**A**, **B** and **C**) and MNs (**D**, **E** and **F**). (A) Streptavidin linked sepharose bead-based pull-downs of decreasing concentrations (lanes 2–9) of purified recombinant 6xHis–Brd4(1–720) with biotinylated 40-bp DNA were separated by SDS-PAGE and visualized by Coomassie Blue staining. The control to rule out non-specific binding is shown with 125 nM 6xHis–Brd4(1–720) minus biotinlyated DNA (lane 10). Molecular weight markers are in lane 1. (B) Experiments were run in triplicate with very similar results with a graphical representation of one gel shown to determine an apparent binding *K_d_*. The intensities of 6xHis–Brd4(1–720) bound to biotinlyated DNA were quantified using ImageJ software and data were fit to the Hill equation. (C) Interactions of indicated Brd4 domains with the biotinylated DNA were measured by the HTRF-based assay. Reactions were run in triplicate with bars representing standard deviations. (D) Nickel bead-based pull-down of purified native MNs with recombinant 6xHis–Brd4(1–720) and immunoblotting with H3 antibodies. A gradient of decreasing MNs (lanes 2–7) was run. Control pull-down is shown with 125 nM MNs without 6xHis–Brd4(1–720) (lane 8) to rule out non-specific binding to nickel beads. Native MNs contain naturally occurring histone modifications and cellular genomic DNA. (E) Graphical representation of immunoblot is also shown to determine an apparent binding *K_d_*. The intensities of H3 bands were quantified using ImageJ software and data were fit to the Hill equation. (F) Nickel bead-based pull-down reactions with indicated fragments of 6xHis–Brd4 and MNs. Bound MNs were detected by immunoblotting with anti-H3 antibody.

We next examined Brd4 binding to DNA (Figure 7A, B and C) and MNs (Figure 7D, E and F). Since Brd4 stimulated the strand-transfer activities of MLV IN (Figure 2), we hypothesized that in addition to binding MLV IN, Brd4 could also bind naked DNA directly. To test this we performed affinity-based pull-down of 6xHis–Brd4 using a biotinylated double-stranded 40-bp DNA fragment as the bait. The results in Figure 7A and B shows that Brd4 can directly bind DNA with a *K_d_* of ∼2.14 µM. These findings help explain Brd4-mediated stimulation of MLV IN strand transfer when naked double-stranded DNA is used as a target (Figure 2). Since target DNA for strand transfer assays and biotinylated 40-bp DNA for binding assays contained random DNA sequences, we conclude that the nature of Brd4 binding to DNA is not sequence specific.

Earlier biochemical and structural studies have shown that BET proteins can also bind modified histone tails. In particular, certain acetylated H3 and H4 peptides but not their unmodified counterparts have been shown to bind individual recombinant BET bromodomains (44,45). However, these interactions exhibited very weak binding affinities with *K_d_*s in the relatively high micromolar range (27,38,46). Since Brd4 binding to naked DNA or cognate-modified histone tails cannot fully explain the tight association of BET proteins with cellular chromatin, we hypothesized that BET proteins may bind with high affinity to native nucleosomes, which contain naturally occurring histone modifications and cellular genomic DNA, through cooperative interactions with both acetylated histone tails and DNA wrapped around core histones. The rationale is supported by our recent studies of LEDGF PWWP, which showed synergetic binding to the H3K36me3 tail and DNA wrapped around the core histones in the context of modified MNs. Results in Figure 7D and E show that 6xHis–Brd4 binding to MNs was much tighter (*K_d_* of ∼61 nM) than its interaction with DNA (*K_d_* of ∼2.14 µM), or the binding of individual Brd4 bromodomains with acetylated peptides (*K_d_* of ∼7 µM for the di-acetylated and ∼215 µM for mono-acetylated H4 peptides) (47).

To delineate the motifs/domains responsible for binding either DNA or MNs, we examined the individual fragments of Brd4 (Figure 1) using a combination of pull-down and HTRF-based assays (Figure 7C and F). Interactions between 6xHis–Brd4 or its fragments to biotinylated 40-bp DNA were monitored by HTRF using an anti-His antibody labeled with the donor fluorophore and streptavidin-europium serving as an acceptor fluorophore with binding detected by a corresponding increase in the FRET signal (Figure 7C). Results show that 6xHis–Brd4 interacts with labeled DNA as expected based on the complementary affinity pull-down data (Figure 7A). When Brd4 fragments were examined, comparable binding was seen for 6xHis-nBrd4(1–461), which consists of both bromodomains as well as the conserved A motif. In addition, we observed DNA binding by 6xHis–Brd4 B/BID(462–599) (Figure 7C). The observed HTRF-signal for DNA binding to 6xHis–Brd4 B/BID(462–599) was slightly higher than with 6xHis–Brd4(1–720) or 6xHis–nBrd4(1–461) possibly due to the closer placement of the donor and acceptor in the bound complex with a much smaller 6xHis–Brd4 B/BID(462–599). When the C-terminal region of Brd4 was examined, the fragments of 6xHis–Brd4 ET(600–678) or 6xHis–Brd4 ET/SEED(600–720) did not have any appreciable binding to DNA.

Next we tested the 6xHis–Brd4 fragments capable of binding to native MNs (Figure 7F), and found that interaction with MNs was seen for 6xHis–nBrd4(1–461) and 6xHis–Brd4 B/BID(462–599), but not for the two C-terminal fragments of 6xHis–Brd4 ET(600–678) or 6xHis–Brd4 ET/SEED(600–720). These results (Figure 7F) corroborate the findings of the DNA-binding assays (Figure 7C) and indicate that the N-terminal region (aa 1–599) of Brd4 binds MNs by engaging both DNA wrapped around the core histones and modified histone tails.

### BET protein-mediated MLV-integration sites positively correlate with acetylated histone marks

**Figure 8.**
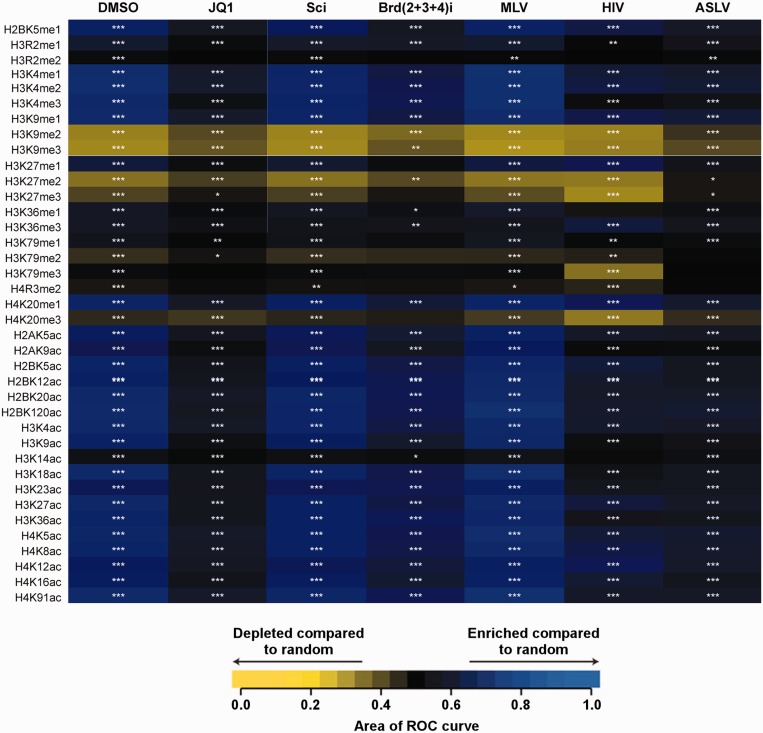
Heatmap summarizing integration frequencies relative to histone post-translational modifications. Integration site datasets are shown in the columns. Histone post-translational modifications are shown in the rows and labeled on the left. The receiver operator characteristic (ROC) curve area method was used to quantify the relationship between the integration site frequencies relative to matched random controls for each of the annotated histone post-translational modification shown on the left. The color key depicts enrichment or depletion of the annotated feature-near integration sites (enrichment is indicated in blue and depletion is indicated in yellow; intensity of the color indicates the strength of the effect). *P*-values are for individual integration site datasets compared to matched random controls, ***P < 0.001; **P < 0.01; *P <0.05. ‘JQ-1’ indicates 500 nM JQ-1 inhibitor treatment, ‘Sci’ indicates scrambled control siRNA knockdown, ‘Brd(2+3+4)I’ indicates siRNA knockdown of all three BET proteins.

We recently demonstrated (21) that inhibiting BET protein binding to chromatin with the small molecule JQ-1 (28), or through concurrent downregulation of the three BET proteins using siRNA, significantly reduced the proportion of MLV integration events at transcription start sites. Here we have extended our analyses of these experimental results (21) to examine potential correlations between MLV-integration sites and various histone marks. The results in Figure 8 show that when compared to matched random controls, MLV integration is significantly enriched at sites near histones containing certain acetylated H3 and H4 tails. This preference is adversely affected by either JQ-1 treatment or treatment with Brd(2+3+4) siRNAs. Comparison to integration site data for HIV-1 and ASLV showed less favored integration near acetylated histone marks. This analysis was further extended to examine if there were statistically significant differences between the test samples (JQ-1 or Brd(2+3+4) siRNAs) versus a control. The pair-wise analysis revealed that the correlations between histone marks and integration sites were significantly different, especially for JQ-1 treatment (Supplementary Figure S4). While statistically significant differences were also observed for siRNA treatment, the lower number of total integrations influenced this analysis. These results correlate closely with MS-based analysis of H3 and H4 posttranslational modifications found at BET protein-associated nucleosomes (48). For example, published results show that BET proteins exhibit strong preference for binding to H3K9ac and H3K18ac but not for H3K14ac (48). Figure 8 shows that MLV integration is enriched in H3K9ac and H3K18ac bound sites, whereas MLV integration is more similar to random at H3K14ac bound sites. Other examples of positive correlations can be seen for BET protein binding (48), MLV-integration sites and the histone marks such as mono-, di- and tri-methylated H3K4 as well as a number of H4 acetylated tails. Conversely some histone modifications including a number of methylated marks at positions H3K9, H3K27, H3K79 and H4K20 are not favored or strongly disfavored for both BET protein binding (48) and MLV integration. Thus results in Figure 8 and published results indicate that BET protein binding to select histone marks determines the distribution of MLV-integration sites on chromatin.

## DISCUSSION

**Figure 9.**
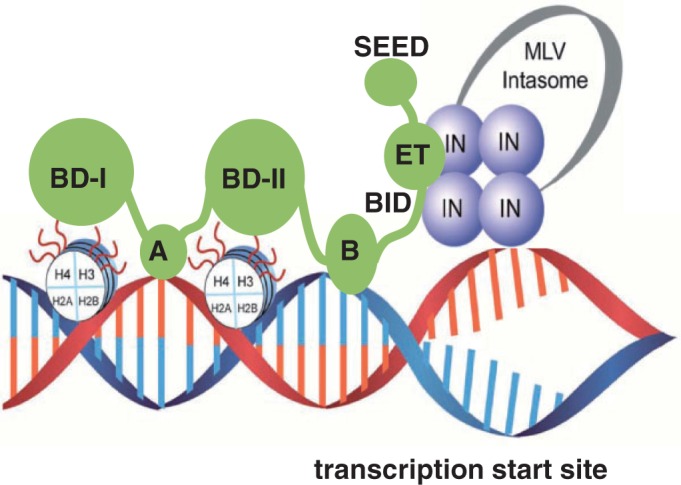
Schematic diagram showing the bimodal mechanism of BET proteins mediated tethering of MLV intasome to select chromatin sites. BET proteins associate with nucleosomes through interactions of BD-I and II with cognate modified H3 and H4 tails as well as motifs A and B binding directly to DNA. The ET domain in the BET proteins directly engages the C-terminal tail of MLV IN to target MLV integration near transcription start sites.

In this report we show that Brd4, which is a representative of the BET proteins (Brd2, 3 and 4), functions as a bimodal tether by establishing complementary high-affinity interactions with MLV IN and MNs (Figure 9). Brd4(1–720) but not its isolated N- or C-terminal fragments effectively stimulate MLV IN strand transfer activities *in vitro*. Furthermore, using complementary MS- and NMR-based approaches we have mapped key interacting interfaces between Brd4 and MLV IN.

We show that MLV IN CTD is both essential and sufficient for high-affinity binding to Brd4. Our findings contrast with recent site-directed mutagenesis studies, which reported that a three amino acid substitution in the CCD of MLV IN compromised its interaction with BET proteins (22); that work did not evaluate the catalytic activities of their mutant MLV INs. Therefore, indirect effects of these mutations on MLV IN–Brd4 interactions have not been ruled out. Here, we demonstrate that both the NTD and the CCD of MLV IN are fully dispensable for binding to Brd4, whereas MLV IN CTD and full-length MLV IN bind Brd4 with very similar, high affinity. MS-based footprinting and truncation mutagenesis studies mapped the key interactions to the C-terminal tail of MLV IN, specifically the last 10 amino acids. We also found that the C-terminal truncation mutants of MLV IN exhibited wild-type catalytic activities *in vitro*, which complements earlier work demonstrating that a deletion of the C-terminal 28 amino acids of IN in the MLV proviral genome did not affect viral infectivity (49). Brd4-stimulated strand-transfer activities of the wild type (∼6-fold) but not for the mutant MLV INs. A recent study has reported that the W391A substitution in full-length MLV IN, which is adjacent to the key interacting interface mapped by the present study, severely impairs its interaction with mouse Brd4 ET (23). It is noteworthy that the C-terminal amino acid tail (aa 391–405) of MLV IN is highly conserved in all gamma-retroviral INs but not in other retroviral INs. Collectively, these findings support the notion that the C-terminal amino acids confer selectivity of BET protein binding for MLV IN.

Our MS-based protein footprinting and truncation mutagenesis studies have identified the ET domain of Brd4 as the binding partner of MLV IN with the BID region also contributing to these interactions. NMR-titration experiments mapped the ET domain residues affected by MLV IN CTD binding and revealed a binding interface on Brd4 ET. Significantly, our NMR studies have also demonstrated that the MLV IN C-terminal tail and in particular the 17 amino acid peptide (aa 389–405) is sufficient for binding to Brd4 ET. Recent site-directed-mutagenesis studies also examined ET residues important for binding to MLV IN and showed that a number of substitutions (Brd2 residues of D687, F688, E689) in the loop connecting helices 2 and 3 impair Brd2 ET domain interactions with MLV IN (22). The corresponding human Brd4 residues (D655, F656 and E657) showed large chemical shift perturbations upon binding to the MLV IN CTD (Supplementary Figures S1 and S2). Site-directed-mutagenesis studies with mouse Brd4 ET have also shown the significance of E652, E654, D656 and E658 (which correspond to E651, E653, D655 and E657 in human Brd4) for binding MLV IN (23). While the limited interactions probed by mutagenesis studies (22,23) do not fully explain the high-affinity binding observed between Brd4 and MLV IN, our NMR results significantly extend the MLV IN CTD-binding interface on Brd4 ET.

The use of NMR has allowed us to examine the entire ET domain to elucidate the residues involved in binding MLV IN CTD. Figure 6C and D show that the majority of Brd4 ET residues affected by MLV IN binding are located on helices 2 and 3. Accordingly, we propose that the MLV IN CTD tail engages helices 2 and 3 (residues colored red in Figure 6C and D) and partly wraps around Brd4 ET to establish additional interactions with the connecting loop between these two helices (residues colored red in Figure 6E and F). These findings thus provide a rational for the high-affinity binding between Brd4 and MLV IN.

We demonstrate that Brd4 binds DNA with a relatively low binding affinity (*K_d_* ∼2.14 µM) but binds more tightly to MNs (*K_d_* ∼61 nM). Published biochemical and structural data studies have shown that isolated recombinant bromodomains of BET proteins preferentially bind certain acetylated H3 and H4 peptides but not their unmodified counterparts (44–46). Yet, the binding affinities for BET protein bromodomains with cognate peptides was in the high-micromolar range (44–46). More recent studies have explored histone peptides containing multiple acetylated sites and demonstrated an increased affinity for such peptides when compared with their counterparts containing only a single modification (47). However, measured affinities for peptides containing multiple acetylated sites were still in the low-micromolar range (the tightest affinity reported to date is *K_d_* ∼7 µM for the BD-I of Brd4 interaction with the di-acetylated H4 peptide (47)); thus, why BET proteins bind tightly to chromatin has remained unanswered.

Our findings that Brd4 binds MNs with significantly higher affinity than naked DNA or isolated acetylated histone peptides suggests cooperative interactions of Brd4 with both protein and DNA components of MNs. These findings are reminiscent of the recently described mechanism for high-affinity binding of LEDGF PWWP with MNs (12). We have determined the NMR structure of LEDGF PWWP and shown that the protein has two distinctive interfaces: a hydrophobic cavity that binds H3K36me3 and the basic surface that non-specifically interacts with DNA. Furthermore, we demonstrated that while binding affinities of LEDGF PWWP with isolated H3K36me3 peptide and naked DNA were in the low-millimolar and low-micromolar range, respectively, the protein bound MNs with high affinity (*K_d_* ∼50 nM) by cooperatively engaging both DNA wrapped around the histone core and cognate histone marks.

Analyses of available structures of BET bromodomains (BD-I and II) shows that BD-Is in all the three BET proteins have a positively charged region (47), which is distinct from its acetylated peptide-binding site and could potentially contribute to DNA binding. While BD-II of Brd2 has a similar positively charged region, BD-IIs from Brd3 and Brd4 do not. More importantly, we found that motifs A and B, which are highly conserved in all the three BET proteins, exhibit a highly basic interface that could play a key role in their interactions with DNA. Again, a parallel can be drawn between these observations and the domain organization of LEDGF/p75, which in addition to the PWWP domain also contains highly basic regions that stabilize its interaction with DNA (50). Thus, cooperative interactions with both DNA wrapped around the core histones and cognate modified peptide tail could be a generic mechanism utilized by many chromatin-binding proteins to ensure their high-affinity-regulated interactions with chromatin.

The ability of BET proteins to preferentially bind select histone marks in turn influences MLV-integration-site selection. For example, there is a positive correlation between certain acetylated H3 and H4 peptides, BET protein-binding sites and MLV-integration sites. Of note, these histone marks are enriched at transcription start sites and near proto-oncogenes. Such observations have significance for the application of MLV-based vectors for human gene-therapy as insertional activation of proto-oncogenes has been implicated in leukemia outcomes in patients treated with MLV-based vectors [reviewed in (51–54)]. Our results in Supplementary Figure S5 show that treatment with small molecule JQ-1, which selectively blocks interactions of BET proteins with cognate histone tails, significantly reduces MLV-integration frequencies near proto-oncogenes. This provides an important proof-of-concept that MLV-integration sites can be altered to potentially reduce adverse side effects and improve the safety of MLV-based vectors used for human gene-therapy.

## SUPPLEMENTARY DATA

Supplementary Data are available at NAR Online.

## FUNDING

U.S. National Institutes of Health [AI062520 to M.K., AI052845 to F.D.B., GM070837 and GM088808 to M.J.R.]. Funding for open access charges: U.S. National Institutes of Health [AI062520].

*Conflict of interest statement*. None declared.

## Supplementary Material

Supplementary Data
