# Supplementary material for: Bimodal high-affinity association of Brd4 with murine leukemia virus integrase and mononucleosomes
Source: Nucleic Acids Res. 2014 Feb 11;42(8):4868–81. doi: 10.1093/nar/gku135 (PMC4005663; doi:10.1093/nar/gku135)
Supplement: Supplementary Data [file supp_42_8_4868__index.html]

Bimodal high-affinity association of Brd4 with murine leukemia virus integrase and mononucleosomes — Bimodal high-affinity association of Brd4 with murine leukemia virus integrase and mononucleosomes — Supplementary Data 

# Bimodal high-affinity association of Brd4 with murine leukemia virus integrase and mononucleosomes

## Supplementary Data

files

**Files in this Data Supplement:**

- Supplementary Data - pdf file
- Supplementary Data - docx file
