# Supplementary figures and images for: Bimodal high-affinity association of Brd4 with murine leukemia virus integrase and mononucleosomes
Source: Nucleic Acids Res. 2014 Feb 11;42(8):4868–81. doi: 10.1093/nar/gku135 (PMC4005663; doi:10.1093/nar/gku135)

A

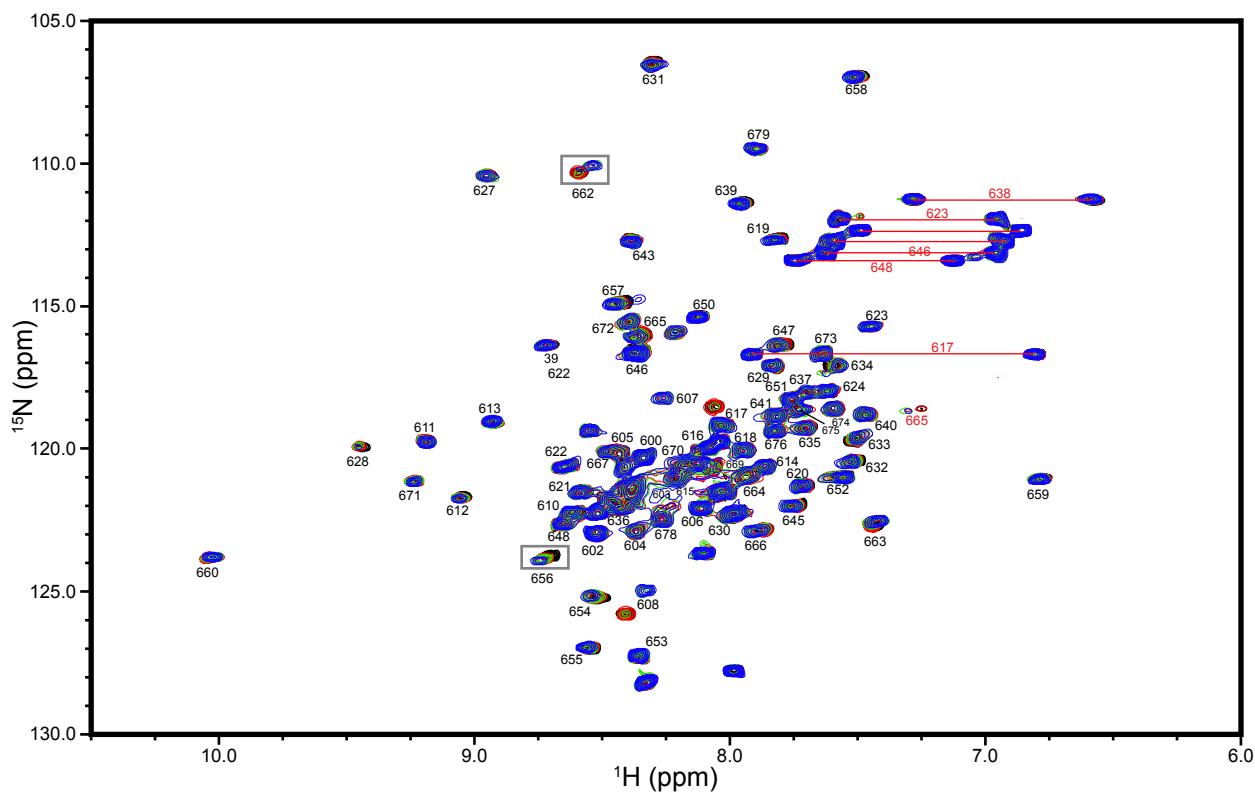

B

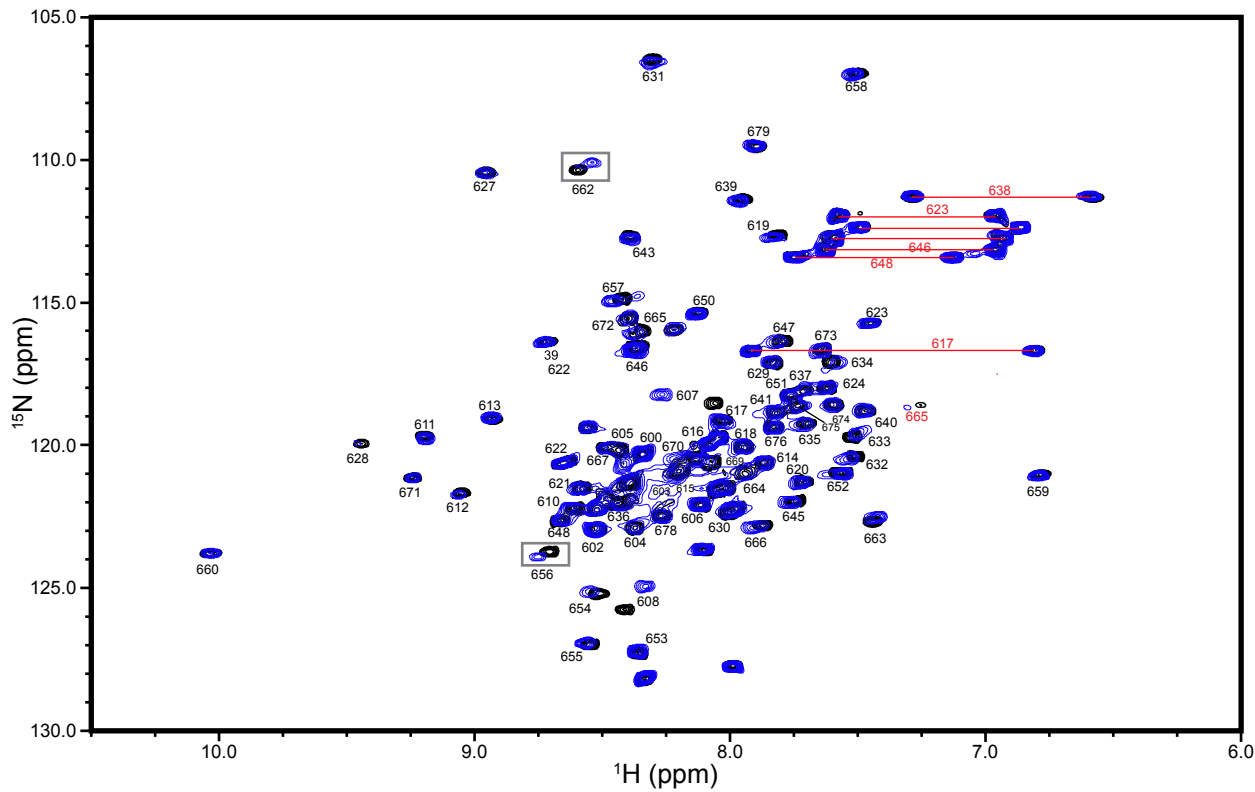

# Chemical Shift Perturbations on Brd4 ET domain induced by MLV CTD

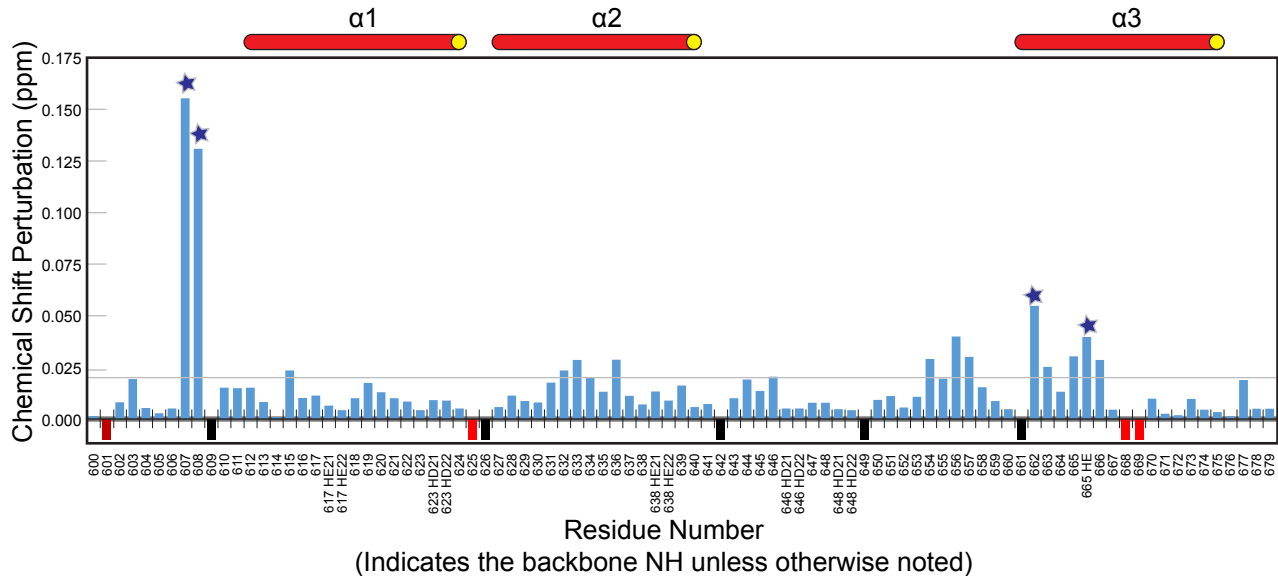

# Arg 665 sidechain Hε

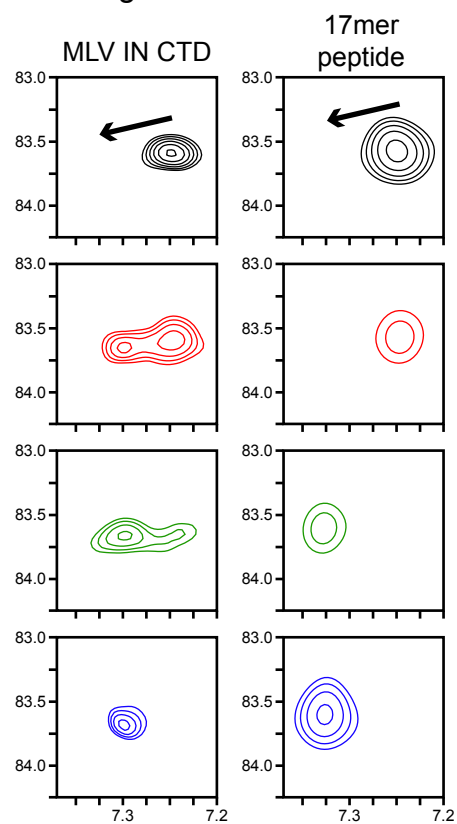

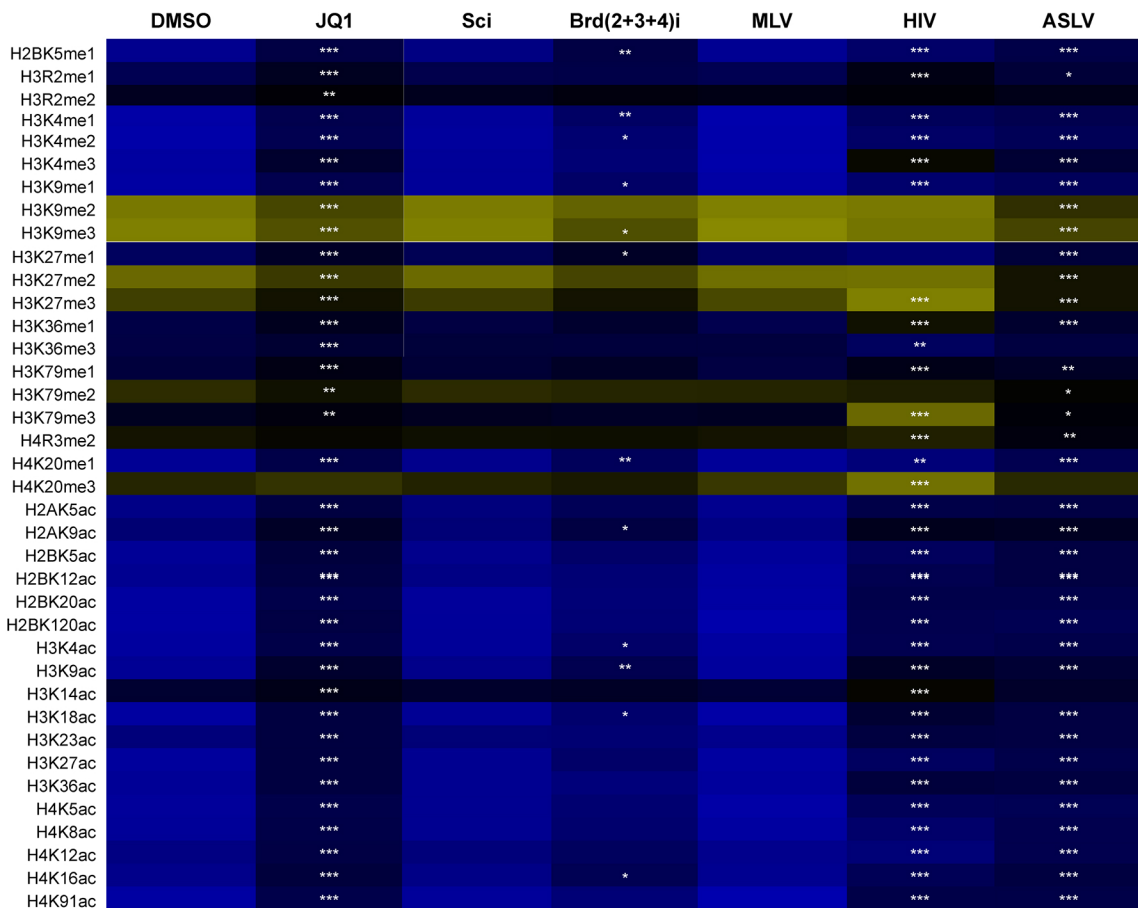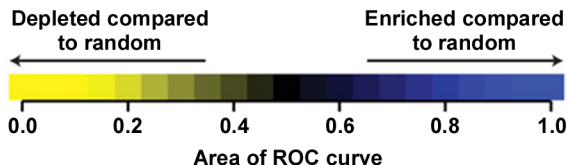

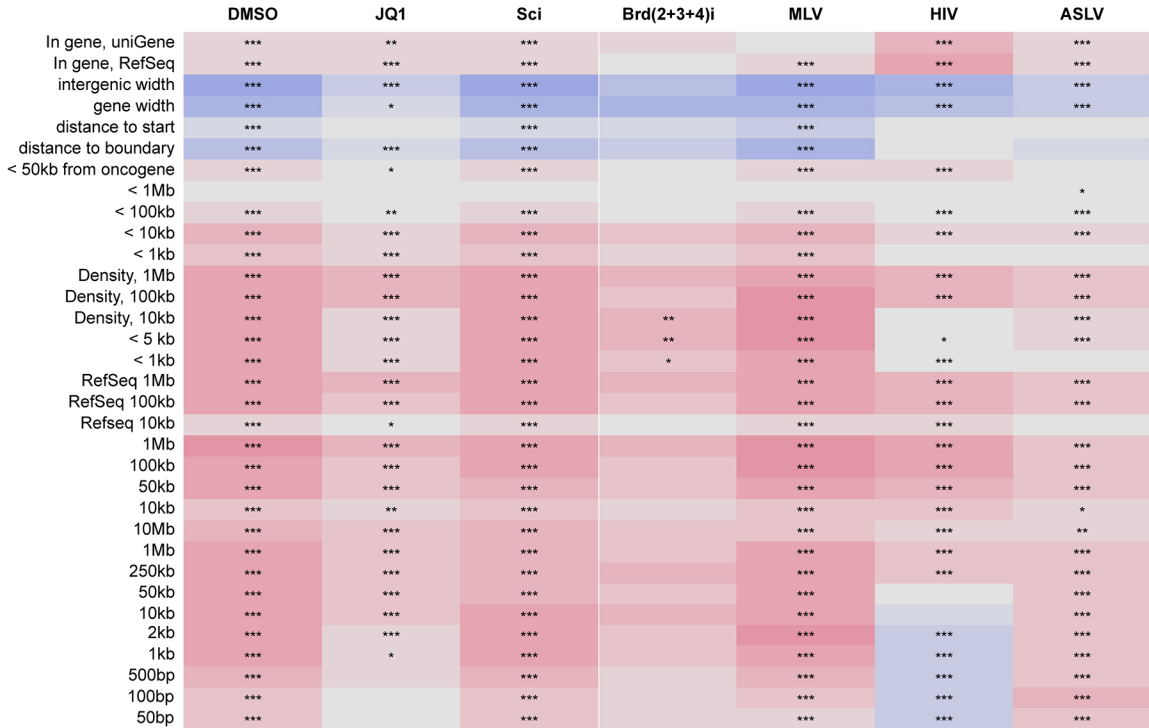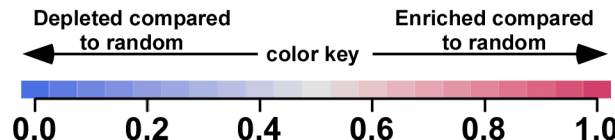

Supplement: Supplementary Data [file supp_gku135_nar-03366-m-2013-File012.pdf]
