# Supplementary material for: Bimodal high-affinity association of Brd4 with murine leukemia virus integrase and mononucleosomes
Source: Nucleic Acids Res. 2014 Feb 11;42(8):4868–81. doi: 10.1093/nar/gku135 (PMC4005663; doi:10.1093/nar/gku135)
Supplement: Supplementary Data [file supp_gku135_nar-03366-m-2013-File011.docx]

**Supplementary materials**

**Supplementary Figure Legends:**

Supplementary Figure 1. NMR titration of MLV IN CTD into ^15^N-labeled Brd4 ET. A) The full titration. 190 μM Brd4 ET in the absence (Black), and presence of 0.5 (Red), 1 (Green), and 1.5 equivalents of MLV CTD (Blue). Backbone amide resonances are labeled in black and sidechain signals are labeled in red. B) The endpoints of the titration. Same coloring used as in A. The grey boxes indicate the residues zoomed in on for Figure 6.

Supplementary Figure 2. Per-residue amide chemical shift perturbations (CSP) on Brd4 ET caused by MLV IN CTD. The CSP given by $\Delta\delta=\sqrt{0.5\left( {\Delta\delta}_{H}^{2}+\left( {{\Delta\delta}_{N}}/5 \right)^{2} \right)}$ (37). Small negative black bars indicate no data due to the presence of a Pro. Small negative red bars indicate no data due to overlap of signals. The secondary structure is mapped above the graph. Resonances exhibiting slow exchange are indicated by a blue star. The grey line indicates the cutoff used in mapping the CSPs to the structure for Figure 6.

Supplementary Figure 3. Perturbation of amide NMR signals of Brd4 ET upon titration with a peptide consisting of 17 amino acids (aa 389-405) at the C-terminal tail of MLV IN. Shown here are the amide resonances from the R665 sidechain Hε. This signal exemplifies the slow exchange regime, which is characterized by slow disappearance of the free peak coupled to the appearance of the bound peak. Top (Black) spectra, unliganded Brd4 ET; second row (Red), Brd4 ET with 0.5 equivalents of peptide; third row (Green), Brd4 ET with 1 equivalent of peptide; bottom row (Blue), Brd4 ET with 1.5 equivalents of peptide. The black arrow indicates the direction of the CSP from the unliganded to the bound state. Resonance assignments are as previously reported (37).

Supplementary Figure 4. Heatmap summarizing integration frequencies relative to histone post-translational modifications. Integration site data sets are shown in the columns. Histone post-translational modifications are shown in the rows and labeled on the left. The ROC curve area method was used to quantify the relationship between the integration site frequencies relative to matched random controls for each of the annotated histone post-translational modification shown on the left. The color key depicts enrichment or depletion of the annotated feature near integration sites (enrichment is indicated in blue and depletion is indicated in yellow; intensity of the color indicates the strength of the effect). P values are for individual integration site data sets of treatments (JQ-1, Brd(2+3+4)i, or MLV) compared to controls (DMSO, Sci, HIV or ASLV respectively), ***P < 0.001; **P < 0.01; *P <0.05. ‘Sci’ stands for scrambled control siRNA knockdown where ‘Brd(2+3+4)i’ stands for siRNA knockdown of all three BET proteins.

Supplementary Figure 5. Heatmap summarizing integration frequencies relative to the genomic features. Integration site data sets are shown in the columns. The genomic features analyzed are shown in the rows and labeled on the left. The size of a given genomic interval used for analysis is indicated by the base pair values shown in the rows. The ROC curve area method was used to quantify the relationship between the integration site frequencies relative to matched random controls for each of the genomic feature shown on the left. The color key depicts enrichment or depletion of the annotated feature near integration sites (enrichment is indicated in red and depletion is indicated in blue). P values are for individual integration site data sets compared to matched random controls, ***P < 0.001; **P < 0.01; *P <0.05.

**Supplementary References:**

37. *Garrett, D. S., Seok, Y. J., Peterkofsky, A., Clore, G. M., and Gronenborn, A. M. (1997) Identification by NMR of the binding surface for the histidine-containing phosphocarrier protein HPr on the N-terminal domain of enzyme I of the Escherichia coli phosphotransferase system. Biochemistry* ***36****, 4393-4398*
